# The Role of Alpha Activity in Spatial and Feature-Based Attention

**DOI:** 10.1523/ENEURO.0204-16.2016

**Published:** 2016-10-05

**Authors:** Rosanne M. van Diepen, Lee M. Miller, Ali Mazaheri, Joy J. Geng

**Affiliations:** 1 Department of Psychiatry, Academic Medical Center, University of Amsterdam, 1105 AZ, Amsterdam, The Netherlands; 2 School of Psychology, University of Birmingham, Birmingham, B15 2TT, United Kingdom; 3 Center for Mind and Brain, University of California Davis, Davis, CA 95616, USA; 4 Department of Neurobiology, Physiology, & Behavior, University of California Davis, Davis, CA 95616, USA; 5 Department of Psychology, University of California Davis, Davis, CA 95616

**Keywords:** alpha, attention, feature, poststimulus, spatial

## Abstract

Modulations in alpha oscillations (∼10 Hz) are typically studied in the context of anticipating upcoming stimuli. Alpha power decreases in sensory regions processing upcoming targets compared to regions processing distracting input, thereby likely facilitating processing of relevant information while suppressing irrelevant. In this electroencephalography study using healthy human volunteers, we examined whether modulations in alpha power also occur after the onset of a bilaterally presented target and distractor. Spatial attention was manipulated through spatial cues and feature-based attention through adjusting the color-similarity of distractors to the target. Consistent with previous studies, we found that informative spatial cues induced a relative decrease of pretarget alpha power at occipital electrodes contralateral to the expected target location. Interestingly, this pattern reemerged relatively late (300–750 ms) after stimulus onset, suggesting that lateralized alpha reflects not only preparatory attention, but also ongoing attentive stimulus processing. Uninformative cues (i.e., conveying no information about the spatial location of the target) resulted in an interaction between spatial attention and feature-based attention in post-target alpha lateralization. When the target was paired with a low-similarity distractor, post-target alpha was lateralized (500–900 ms). Crucially, the lateralization was absent when target selection was ambiguous because the distractor was highly similar to the target. Instead, during this condition, midfrontal theta was increased, indicative of reactive conflict resolution. Behaviorally, the degree of alpha lateralization was negatively correlated with the reaction time distraction cost induced by target–distractor similarity. These results suggest a pivotal role for poststimulus alpha lateralization in protecting sensory processing of target information.

## Significance Statement

A substantial amount of research has been dedicated to elucidating the role of alpha oscillations in preparation of upcoming targets and distractors. Considerably less research has focused on alpha activity after presentation of those stimuli. Using a novel paradigm, in which spatial attention was manipulated using informative and uninformative spatial cues and feature-based attention using distractors that varied parametrically in color-similarity to the target, we show that poststimulus alpha hemispheric lateralization protects target processing after spatial and feature-based target selection. Modulations in alpha power can therefore be regarded as a general mechanism for direction of proactive and reactive attention.

## Introduction

Attention allows us to selectively process information that is relevant to our current goals and suppress irrelevant distractors. A number of studies have found a link between the allocation of preparatory selective visuospatial attention and hemispheric modulations of oscillatory activity in the alpha range (8–12 Hz) over occipital regions of the cortex. The power of alpha activity has been found to decrease over occipital regions contralateral to target presentation versus when the target was expected ipsilaterally (Worden et al., 2000; Kelly et al., 2006; Thut et al., 2006; Kerlin et al. 2010). This lateralization is believed to result in enhanced processing of targets (Osipova et al., 2008; Weisz et al., 2011; Bauer et al., 2012; Lange et al., 2013) and decreased processing of distractors in downstream areas (Zumer et al., 2014). In the current study, alpha activity is examined pre- and poststimulus presentation during spatial and feature-based target selection.

Whereas there is clear evidence for the role of alpha in spatial attention, its involvement in feature-based attention remains relatively unclear. Previous research has shown that mechanisms of feature-based attention prioritize target-related features in a spatially nonspecific way, even when the location of the target is known (Saenz et al., 2002; Serences and Boynton, 2007; Andersen et al., 2008; Zhang and Luck, 2009). However, none of those studies have examined whether alpha oscillations in response to a spatially cued target can protect against attentional capture by a feature-based distractor. In terms of hierarchical processing in the visual system, it is clear that spatial attention operates through selection at lower (retinotopically mapped) levels of neuronal representations. Conversely, attentional selection of specific features can occur only in functionally specialized cortical regions (e.g. Snyder and Foxe, 2010). This raises the question of how higher-level featural attention depends on spatial attention and vice versa. It could be the case that both spatial and featural attentional mechanisms operate together by boosting signals from the attended (retinotopic) location, or it may be that each operates separately at the level of its specialized representations. Conversely, spatial attention may itself be deployed in a way that is sensitive to feature-based attention, suggesting an interaction between the two.

If mechanisms of feature-based selection recruit spatial attention once a target is identified, then we might expect the presence of lateralized alpha even when a target can be selected based on color only. Previous work has suggested that selection of an object based on features is followed by a shift of spatial attention (Öğmen and Breitmeyer, 2006). Finding lateralized alpha during feature-based selection would be novel evidence that spatial attention is recruited to suppress distractor processing and shield target processing, once a target has been identified based on its nonspatial features. In contrast, if the mechanisms of spatial and feature-based attention are largely independent, then we would not expect effects of spatial attention associated with posterior alpha to impact feature-based attentional capture, nor would we find changes in alpha activity when spatial cues are absent.

The purpose of the current experiment was to explore how spatial attention and featural attention might interact. We used spatial (hemispheric) lateralization of alpha suppression to index the physiological correlates of spatial attention and asked whether this neurophysiological signature depended on attentional set for features. We used a novel visual cuing paradigm involving the presentation of a bilateral visual search array; spatial attention was manipulated with a valid precue, and feature-based attention was manipulated by the degree of distractor-to-target color similarity. Behavioral responses were collected simultaneously with ongoing electroencephalography (EEG) that measured posterior alpha as an index of spatial attentional selection. Although previous studies have focused exclusively on the modulation of alpha power during the cue–target interval, we also examined how post-target alpha activity was modulated as function of the target-similarity of distractors.

In addition to alpha oscillations, we examined potential post-target differences in theta activity. Increased theta-band activity in midfrontal channels has been found in situations of conflict and resolution processes (Cavanagh et al., 2012; Nigbur et al., 2012; Cohen and Donner, 2013; Cavanagh and Frank, 2014; Cohen, 2014; Van Driel et al., 2015) and could therefore be an indication of the absence of selective attention occurring in visual cortex.

In brief, we show a profound interaction in alpha lateralization and a complementary modulation in theta activity, suggesting that attentional gain at lower (retinotopic) levels of the visual hierarchy is mediated by convergent spatial and feature-based mechanisms.

## Materials and Methods

### Participants

Twenty healthy participants (14 female) were recruited from the University of California Davis. The average age of included participants (*n* = 16, see Methods, Analyses, Behavioral) was 24.8 years, and all were right-handed. Participants reported normal or corrected-to-normal vision, no color blindness, and no history of neurological or psychological illness. Before the start of the experiment, signed informed consent was obtained in accordance with the University of California Davis Institutional Review Board.

### Procedure

The stimuli were presented using Presentation software (Neurobehavioral Systems, Albany, CA) on a 24-inch monitor with a refresh rate of 60 Hz. Before the start of the experiment, participants practiced 60 trials or more until they reached a threshold of 70% correct. Participants were seated approximately 100 cm from the monitor.

### Task

Participants reported the identity of a letter (A, B, or C) presented in a target stimulus by making three-button alternative forced choice decision using their right hand. The target was a red diamond that appeared on the left or right of a central fixation cross with equal probability. The distance from the fixation cross to object center was 5.5 degrees of visual angle; the width of the object from center to edge was 1.1 degrees.

A distractor was presented in a square in the hemifield opposite to target. The distractor also contained a letter (A, B, or C), but the letter was never the same as the target. The color of the distractor was the same red as the target (D1, luminance cd/m^2^, and CIE *x*,*y* = 48.8, 0.639, 0.343) or was one of three other colors ranging from orange to yellow (D2 = 52.7, 0.635; 0.343; D3 = 73.4, 0.578, 0.385; D4 = 110, 0.510, 0.385). The brightest object based on luminance values was the most yellow distractor. Thus, luminance and target similarity were opposite to each other, and the effect of one on attention cannot explain the effect of the other. To ensure that participants did not become overtrained on shape selection, the target and distractor shape were switched every block (e.g., the square became the target and the diamond became the distractor). The target and distractor colors remained the same. Thus, although it would be possible for the subject to select the target entirely based on shape, color was never irrelevant. The target could be selected based on color on 75% of trials (D1 occurred on only 25% of trials), and whereas the target color was consistent throughout the experiment, the target shape changed from block to block (every 48 trials). This design increased the likelihood of subjects continuing to use color as a criterion for target selection, despite shape also being indicative. The behavioral results also provide evidence that mechanisms of feature-based attention for color led to greater selection of distractors as a function of target-color similarity.

In 50% of the trials, a spatial cue (< or >) indicated the location (left or right) of the upcoming target 1200–1500 ms before visual search display with 100% validity (i.e., cued trials). On these trials, it was possible for participants to anticipate the target location. In the other 50% of trials, the cue (◊) was uninformative about the location of the target (i.e., uncued trials). On uncued trials, participants identified the target based on shape and color. Participants were asked not to make an eye movement toward the target but to shift their attention covertly. Fixation was monitored using an EyeLink 1000 Desktop Mount (SR Research) eye-tracker acquiring data at 500 Hz from the right eye.

**Figure 1.**
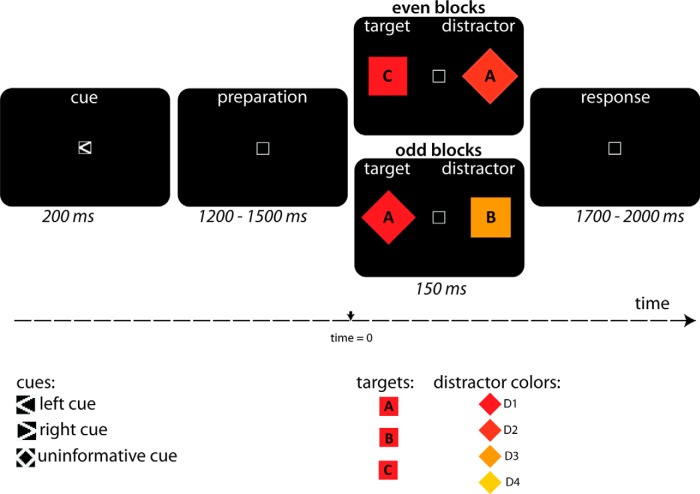
Task procedure (not at scale). The trial started with a 200-ms spatial cue. An informative cue indicated whether the target would appear on the right or left of the fixation box with 100% validity. An uninformative cue indicated that the target could appear at either side with 50% chance. Then participants had 1200–1500 ms to prepare for the upcoming target. A target and distractor were shown for 150 ms, after which participants made a three-alternative-forced-choice response based on whether the target object contained the letter A, B, or C. After 1700–2000 ms, the cue for the next trial was presented. The distractor also contained a letter, but never the same letter as the target. (Importantly, because there were three possible letters, guessing the target identity based on the distractor letter would produce 33% correct performance.)

An example sequence of a trial is illustrated in Figure 1. First, the cue (left, right, uninformative) was shown for 200 ms. A variable cue–target interval lasting 1200–1500 ms followed the cue to reduce temporal expectancies for the onset of the search screen. The target and distractor were shown for 150 ms, followed by another variable target–cue interval, during which participants responded to the target. Every condition (2 cue × 4 distractor-similarity) included 108 trials, for a total of 864 trials, lasting about 48 min. Trials were pseudorandomized such that all possible trial types (distractor-type × cue-presence × lateralization × target letter) were presented before repeating trials. Breaks occurred every 48 trials.

### EEG acquisition and processing

EEGs were acquired using a 64-channel cap system with a 10/20 layout developed by Biosemi (Amsterdam, Netherlands). The EEG was sampled at 1024 Hz and rereferenced to Cz during importation into Matlab (MathWorks, Natick, MA) using EEGLAB 11 (Delorme and Makeig, 2004).

Using EEGLAB the EEG, data were rereferenced to average reference, high-pass-filtered at 0.5 Hz, and epoched from –2.4 to +1.9 s, time-locked to target onset. Epochs were baseline-corrected using –0.2 to 0 s pretarget. Incorrect trials and trials with an eye movement during the cue–target interval were removed from further analyses (see Methods, Analyses, Behavioral). Remaining ocular artifacts were removed using independent component analysis (ICA, infomax algorithm) incorporated as the default “runica” function in EEGLAB. Principle component analysis (PCA) was used to reduce dimensionality of the data before performing ICA. Then data were transported into Fieldtrip 13_1_1b (Oostenveld et al., 2011) format. A semi-automatic routine (using the “ft_rejectvisual” function of Fieldtrip) was applied to the EEG data to remove epochs with noise.

### Time frequency representation

Time frequency representations (TFRs) of power were estimated per trial, using sliding time windows tapered with a Hann window having an adaptive time window of three cycles for each frequency of interest (Δ*T* = 3/*f*). Similar approaches were used by Osipova et al. (2006), Jokisch and Jensen (2007), Mazaheri et al. (2009), and Van Diepen et al. (2015). Although this approach uses a smaller number of cycles in comparison to Morlet wavelet approaches of time frequency decompositions (i.e., 5–7 cycles); it does afford the maximum temporal resolution for pre-and-post fluctuations in alpha power. A baseline correction was applied such that every time point represents the relative change in power from the average power at baseline [(power time point – power baseline)/power baseline]. A 400-ms interval before cue presentation was used as the baseline interval, ending 100 ms before the possible onset of a cue with the largest cue-to-target interval.

### Analyses

#### Behavioral

Trials were rejected when the target was not identified or when participants moved their eyes toward the target or distractor. An eye movement was identified as a horizontal eye movement exceeding 1.5° from central fixation during the interval from cue presentation to the response. The quality of eye-tracker data of five participants was not sufficient to detect eye movements. For three of those participants, an electro-oculogram was recorded at the outer canthi of the left and right eyes, and eye movements were identified by visual inspection of the data. On average 7.1 ± 1.5% of trials were removed due to eye movements. Eye movements were only removed using ICA for the two participants without eye data. (Results were similar when the analyses were repeated without these two participants.)

Participants scoring two standard deviations below average accuracy were excluded from analyses (two participants). In addition, the presence of a distractor-similarity effect was assessed for every participant by applying an independent-samples *t* test between the reaction times (RTs) for trials with the most similar distractor (D1) and from the least similar distractor (D4), using correct trials only. Data of two participants were removed because they did not show a basic feature-based distractor similarity effect (*p* < 0.05; i.e., the target-colored distractor did not interfere more with target processing than a distractor from a different color category). This was surprising, and suggested these subjects were atypical given that the effect was expected from decades of psychological research (e.g., Treisman and Gelade, 1980; Duncan and Humphreys, 1989; Wolfe and Horowitz, 2004). Note, however, that this exclusion criterion was entirely based on the main effect of feature-based attention and was orthogonal to our analyses of interest on the interaction between spatial cueing and feature-based attention. A second independent-samples *t* test confirmed that all remaining participants showed a significant reduction in RT in trials with an informative cue (see Results, Behavioral); hence no data had to be excluded based on the absence of a spatial cuing effect.

The effect of condition on reaction times and percentage of correct responses was calculated using a two-way repeated-measures ANOVA with two factors: cue presence (cued/uncued) and distractor color (D1–D4). Furthermore, we examined for every condition whether participants scored above chance level by comparing the score against chance level (33.4%), using an independent-samples *t* test (two-tailed, Bonferroni correction for multiple comparisons).

#### EEG

To increase signal-to-noise ratio, data were pooled over hemispheres by flipping the sources with respect to experimental condition, according to the procedures outlined in Buchholz et al. (2013, 2014). Data from trials with left targets were mirrored and pooled with data from right trials. This created a dataset in which the right hemisphere denotes activity ipsilateral to target presentation and the left hemisphere represents contralateral activity.

#### Statistical

Changes in alpha power (averaged across 8–12 Hz) between conditions were statistically assessed by means of cluster-level (channels and time points) randomization (Maris and Oostenveld, 2007) procedure. This test controls the Type I error rate involving multiple comparisons (e.g. multiple channels or time-frequency tiles). A probability value here is obtained through the Monte Carlo estimate of the permutation *p*-value of the cluster of channels by randomly swapping the conditions in participants 1000 times and calculating the maximum cluster-level test statistic. A similar procedure has been used in a number of previous studies (Jokisch and Jensen, 2007; Mazaheri et al., 2009).

#### Feature-based attention

**Figure 2.**
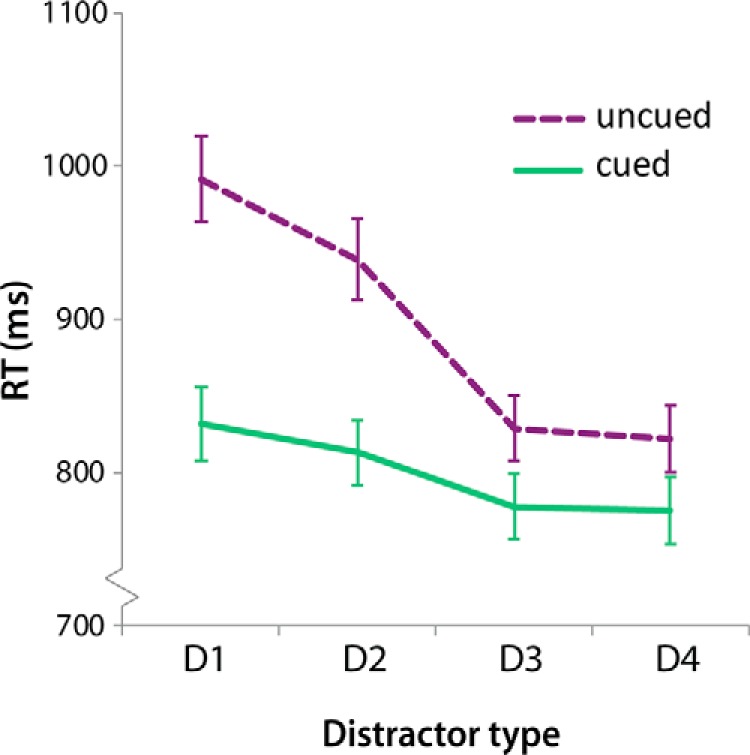
Mean reaction times (+ SEM) as a function of distractor-type for cued and uncued trials. Uncued trials show that increased target-distractor similarity (D1) slows down RTs. This effect was reduced when a spatial cue indicated target location before presentation.

Trials with distractor similarity levels of D1 and D2 were pooled into a “high distraction” condition, and trials with distractors D3 and D4 were pooled to create a “low distraction” condition. The decision for these groupings was driven by the reaction time performance of the participants (Fig. 2). The resulting conditions were tested individually for alpha lateralization after presentation of the visual search display. As described above, a cluster-based permutation test was performed for every condition, contrasting ipsilateral to contralateral activity for all time points between 0 and 1500 ms. Reported results show topographies and statistics after averaging time points containing a cluster of electrodes with *p* < 0.05 (Monte Carlo corrected for multiple comparisons).

#### Behavioral consequence of lateralization during feature-based attention

The relationship between the alpha lateralization and task performance was assessed by correlating the difference in reaction times between targets with low similarity distractors and those with high similarity distractors, with the posterior alpha lateralization across the participants.

The obtained “distractor cost” represents the behavioral consequence of increased distraction. Only uncued trials were used, so cue-related spatial attention did not affect the analysis. Alpha lateralization was calculated by subtracting the average alpha power contralateral to target presentation from alpha activity ipsilateral to target presentation. Therefore, oscillatory power was averaged over channels and time points, showing a significant difference in lateralization between trials with low and high target–distractor similarity. The difference in lateralization between low and high similarity distraction for every participant was correlated with the individual distractor cost using Spearman's rank-order correlation.

#### Frontal theta

A difference in theta activity (averaged across 4–6 Hz) between the low and high distraction condition was also examined for every time point between 0 and 1500 ms. Theta activity was averaged over time points containing a cluster with *p* < 0.05. Trials with left and right targets were pooled without transposing the data to an ipsilateral and contralateral set. Note that we are assuming here that feature-based attention does not show any (spatial) lateralization, enabling us to pool data from both hemispheres. The length of the sliding time window used to calculate the power of a theta oscillation is wider than for an alpha oscillation (3/*f*). The baseline was therefore changed to the pretarget period (–700 to –300) to avoid spurious results due to temporal leakage from the cue or post-target activity. Because only trials with uninformative cues were contrasted and left and right target trials were grouped, this pretarget period should not show any differences between conditions.

## Results

### Behavioral

The mean RT for correct trials was 847.4 ± 21.6 ms. The RT data were entered into a two cue presence (cued, uncued) × four distractor similarity (D1–4) repeated-measures ANOVA. A main effect of cue [*F*(1,15) = 85.4, *p* < .0001] was found such that RT was shorter for informative cue trials, 799.3 **±** 21.7 ms, than for, uncued, trials, 895.5 **±** 22.6 ms. Participants were thus able to use the cue to select the target faster (Fig. 2). When distractor conditions were examined separately, all distractor conditions showed a benefit from cue presence (*p* < 0.001 for cued vs. uncued trials, two-tailed, Bonferroni corrected for multiple comparisons).

A main effect of distractor similarity was also present [*F*(3,45) = 67.9, *p* < .0001]. Distractors induced more distraction when the color was more similar to the target color. Trials with the highest target-distractor similarity (D1) showed the longest reaction times, 910.2 ± 24.6 ms. Reaction times decreased when distractor colors became less similar to the color of the target (D2, 875.2 ± 22.5; D3, 803.6 ± 21.0; D4, 798.8 ± 21.2 ms). Post hoc tests were significant (*p* < .0001, Bonferroni corrected for multiple comparisons) for all possible pairs except D3 and D4.

Most importantly, the interaction between the spatial cue and distractor-similarity was significant [F(3,45) = 34.8, *p* < .0001], suggesting that the spatial cue facilitated target selection to a different extent for the different distractor colors. The difference in reaction times for cued and uncued trials was largest for the most similar colors (D1, 159.8,1 ± 16.5 ms) and decreased as distractors became less similar in color (D2, 126.3 ± 17.3; D3, 51.5 ± 7.5; D4, 46.9 ± 8.5 ms). Pairwise comparisons using a Bonferroni correction show that the amount of cue-related benefit (RT uncued – RT cued) was significantly different between all distractor conditions (*p* < 0.049), except for D3 and D4 (*p* = 1.000).

The presence of a valid cue facilitated target detection when the distractor was highly similar to the target, but the usefulness of the spatial cue was reduced when the target was highly distinct from the distractor and competition for attention during visual search was low. Thus, the spatial cue mainly facilitated behavior when feature-based competition for attention during visual search was high. Based on these RT results, the distractor conditions that were most distracting (D1 and D2) and the least distracting (D3 and D4) were collapsed together in subsequent analyses and labeled as high-similarity and low-similarity distractors, respectively.

Average accuracy was 87.9 ± 9.7%. Performance decreased when distractors were more similar to the target [main effect of distractor type, *F*(3,45) = 3.249, *p* = 0.016]. No difference in performance was present between cued and uncued trials [no main effect of cue presence, *F*(1,15) = 1.274, *p* = 0.277]. However, an interaction was present in accuracy scores between cue presence and distractor type [*F*(3,45) = 3.175, *p* = 0.033], indicating that cues increased accuracy only for trials with high target-distractor similarity. Furthermore, all participants scored above chance for every condition (33.4% based on three target letter identities, or 50% based on guessing after knowing what letter is in the distractor). *t* tests show that scores for all conditions significantly differ from chance (*p* < 0.0001 for all comparisons, corrected for multiple comparisons).

Together, these data demonstrate that the effect of distractor similarity was attenuated when a valid spatial cue was presented, especially when the distractor was very target similar. This suggests that participants used the spatial cue to preselect the target location and that doing so protected them against attentional capture by the feature-similar distractor. The spatial cue affected behavioral performance to a lesser extent when the distractor color was dissimilar, mostly likely because feature-based competition was low and performance was already at, or near, ceiling.

### EEG

#### Informative spatial cues induce alpha lateralization in posterior channels

**Figure 3.**
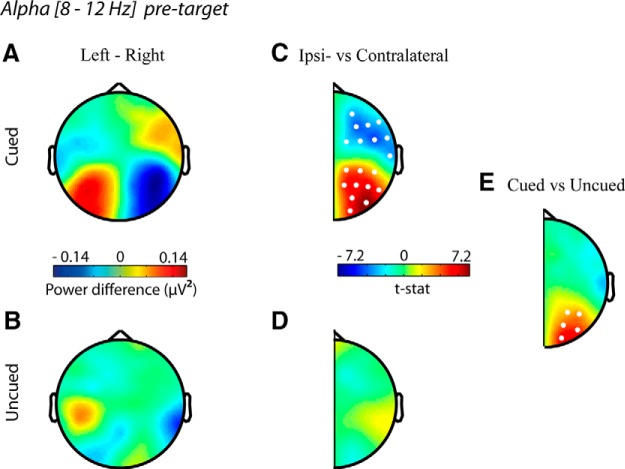
Scalp topography of pretarget lateralization in alpha activity (averaged across 8–12 Hz and time –1050 to 0 ms). (***A***,***B***) The grand average power of alpha activity for left minus right targets. (***C***,***D***) Data combined across hemispheres and compared for differences between channels located contralateral and ipsilateral to target presentation. Warm colors indicate that alpha power was higher contralateral to distractor presentation (i.e., ipsilateral to target presentation). (***E***) The lateralization was significantly different between cued and uncued trials for all time points between –1050 and 0 ms before target presentation. White dots represent channels that showed a significant difference (*p* < 0.05) using the cluster-based permutation test.

We first conducted an analysis on the main effect of cue presence to replicate previous studies demonstrating alpha lateralization in response to a spatial cue. As expected, we found that after presentation of a cue signaling an upcoming left or right target, alpha activity in channels located contralateral to the distractor increased compared with channels contralateral to the target location (Fig. 3*A*,*C*; –1500 to 0 ms pretarget, *t* = 27.23, *p* < 0.001, Monte Carlo *p*-value, corrected for multiple comparisons). The modulation was absent when left and right target trials with uninformative cues were contrasted (Fig. 3*B*,*D*; no clusters to report). A significant interaction in alpha lateralization was present between cued and uncued trials from –1050 to 0 ms before target onset (Fig. 3*E*; *t* = 19.94, *p* = 0.008, Monte Carlo *p*-value, corrected for multiple comparisons). This demonstrates that participants did indeed attend to the cued location in this study and that doing so produced a difference in lateralized alpha.

#### Cue-induced alpha lateralization reduces feature-based distractor competition

**Figure 4.**
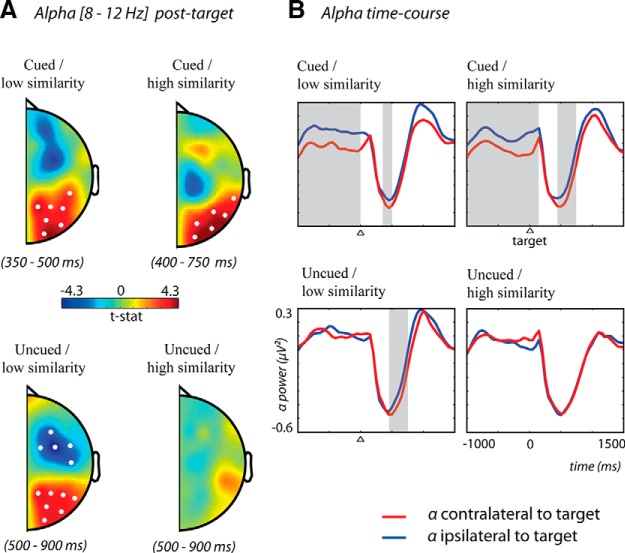
Scalp topographies and time course of alpha activity (averaged over 8–12 Hz) showing lateralization in alpha activity after presentation of the target and distractor. (***A***) Data combined across hemispheres and compared for differences between channels located contralateral and ipsilateral to target presentation. Warm colors indicate that alpha power was higher contralateral to distractor presentation (i.e., ipsilateral to target presentation). White dots represent channels that showed a significant difference (*p* < 0.05) using the cluster-based permutation test. (***B***) Time courses of alpha activity for ipsilateral (blue) and contralateral (red) to target presentation. Gray shaded areas represent time intervals where a significant lateralization was present as found by the cluster-based permutation test. Trials with an uninformative cue and high distraction were the only condition in which no lateralization of post-target alpha activity was present.

In addition to preparatory changes in alpha activity, we also examined alpha activity after the visual search display was presented. In all conditions except when the cue was uninformative and target-distractor similarity was high, alpha activity was significantly lateralized such that it was lower over regions contralateral than ipsilateral to the target (Fig. 4). Spatially cued trials with low-similarity distractors demonstrated this lateralization from 350 to 500 ms after target onset (*t* = 23.68, *p* = 0.003, Monte Carlo *p*-value, corrected for multiple comparisons); cued trials with high-similarity distractors from 0 to 150 ms and reappearing again at 450 to 750 ms after target onset (*t* = 21.85, *p* = 0.002, Monte Carlo *p*-value, corrected for multiple comparisons); and uncued trials with low-similarity distractors from 500 to 900 ms post-target (*t* = 22.71, *p* = 0.004, Monte Carlo *p*-value, corrected for multiple comparisons). Although there was significant alpha lateralization in these three conditions, note that the onset of alpha lateralization in all conditions was relatively late after stimulus onset; moreover, the onset in the cued conditions occurred earlier than the uncued condition. This suggests that alpha lateralization may reflect spatially lateralized attentive processing, rather than the initial selection of a particular object. Additionally, the fact that this process was later in the uncued condition is consistent with the idea that spatially selective processing of the target (and suppression of the distractor) was preceded by the selection of the target based on its color.

It should be noted that eye movements were eliminated and the target and distractor were clearly lateralized with respect to fixation throughout the trial. Therefore lateralized alpha reflected the degree to which attention was consistently oriented toward the target and away from the distractor during visual search. Clear lateralization suggested that the target was consistently selected with little attention to the distractor, whereas a lack of alpha lateralization suggested that the distractor competed successfully to capture attention as frequently as it did not, on average.

These results are consistent with the RT data and provide further evidence that the spatial cue was used to successfully select the target and suppress the distractor, even in the presence of strong competition from a high-similarity distractor. Similarly, alpha lateralization in the uncued low-similarity condition confirms that spatial attention was easily drawn to the target because competition for attention based on feature similarity was low. However, trials with an uninformative cue and a high-similarity distractor did not show any lateralization. This was even true when the statistical threshold was decreased, when a pretarget instead of precue baseline was used, and when activity was averaged over time (same time interval as lateralization was found for uncued low-distraction trials) to increase the signal-to-noise ratio. This result is consistent with the RT data in indicating that the only condition in which the distractor competed successfully against the target for attention was when there was no spatial cue and distractor similarity was high.

#### Individual variability in post-target alpha lateralization correlates with RT distractor cost on uncued trials

**Figure 5.**
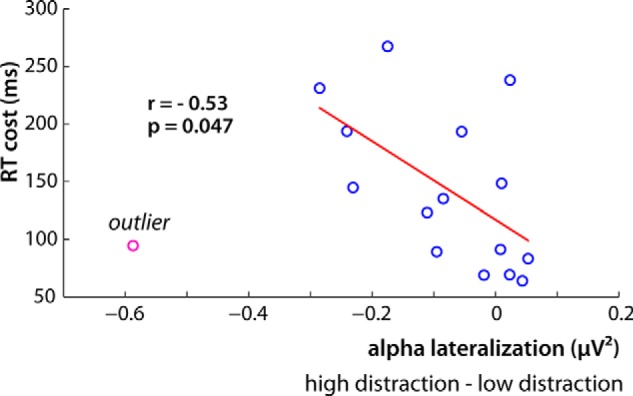
Correlation between the difference in post-target alpha lateralization and the cost in RT for uncued trials. Participants with a larger difference in alpha lateralization between the high and low distraction showed a greater RT cost for high distraction trials. An outlier is shown in pink.

Together, the previous RT and EEG results suggested that greater alpha lateralization during visual search was associated with less distractor interference. Next, we tested whether this relationship predicted individual differences in behavioral RT due to distractor similarity in the uncued conditions. To do so, we created an index of distractor cost (RT high-similarity minus RT low-similarity) and an index of difference in alpha lateralization (lateralization in high-similarity minus low-similarity). Note that less lateralization in the high-similarity condition relative to the low-similarity condition produces larger negative values. Inspection of the data indicated a single outlier (Fig. 5; pink data point). Removal of the outlier resulted in a significant correlation of *r* = –0.53, *p* = 0.047, indicating that participants with greater RT costs in the high-similarity condition also had less alpha lateralization in the high-similarity condition relative to the low-similarity condition (Fig. 5). To be conservative, we also ran the correlation with the outlier, which produced marginally significant correlation, *r* = –0.45, *p* = 0.082. This result is consistent with the group data in showing that alpha lateralization was indicative of less distractor interference, and moreover, that the magnitude of the RT cost within an individual was correlated with the degree of alpha lateralization in the high-similarity compared with low-similarity conditions. This suggests a direct relationship between poststimulus alpha lateralization and the degree of distraction produced by a high-similarity distractor.

#### Post-target midfrontal theta increases for highest distraction

**Figure 6.**
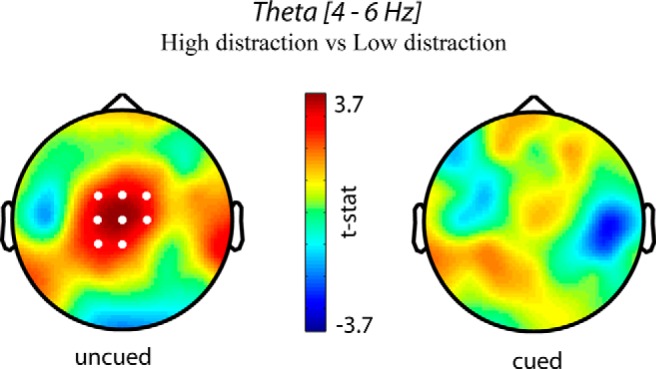
Scalp topographies showing a relative increase in post-target theta activity (averaged across 4–6 Hz and time 700–1250 ms) for trials with high target-distractor similarity when no preceding cue was presented (left). White dots represent channels that showed a significant difference (*p* < 0.05) using the cluster-based permutation test. When a spatial cue was presented before target presentation (right), no difference in theta was found between the low and high target-distractor similarity conditions.

Our previous results suggest that a greater alpha lateralization translates to a better target selection, and this was found at the group level between conditions as well as at the individual level for uncued trials. These results indicate that alpha lateralization is a reliable index of target selection and distractor suppression. Only one condition did not show significant alpha lateralization, and this condition also produced the longest RTs, indicating a failure to consistently suppress attention to the high-similarity distractor. However, inference regarding the presence of distractor competition in that condition was based on a null result: that is, nonsignificant alpha lateralization. To further examine the hypothesis of increased attentional competition in this condition, we compared theta activity between the two uncued conditions (low-similarity vs. high-similarity distractors). If the lack of alpha lateralization in the high-similarity condition is due to greater distractor competition, we would expect a concomitant increase in midfrontal theta as a reflection of greater reactive attention control. Consistent with our expectations, the uncued trials with high-similarity distractors did indeed show an increase in average theta activity (4–6 Hz) in midfrontal channels in the time window 700–1250 ms after target onset compared with the low-similarity condition (*t* = 24.40, *p* = 0.013, Monte Carlo *p*-value, corrected for multiple comparisons). No such difference was present between the cued conditions (Fig. 6; no cluster to report). This is the opposite of the alpha lateralization found between these same conditions in the previous analysis. The result is consistent with the conclusion that the lack of posterior alpha in the uncued high-similarity condition was due to increased distractor competition.

## Discussion

Shifting attention to a particular location in space allows observers to detect objects in the attended location better than in unattended locations (Helmholtz, 1867; Posner, 1980). This effect is supported by increases in the gain of sensory neurons with receptive fields over the attended location (Hillyard et al., 1994; Hopfinger et al., 2000; O’Connor et al., 2002). In contrast, attentional capture by task-relevant features is spatially nonspecific. For example, target-colored objects capture attention even if they appear in unattended, or even task-irrelevant, locations (Serences and Boynton, 2007; Andersen et al., 2009; Zhang and Luck, 2009) . Thus, feature-based attention is thought to operate in parallel across the visual field, leading to attentional capture by target-similar distractors (Folk et al., 1992). However, it remains unclear whether feature-based attentional capture interacts with spatial attention.

In the current visual search study we explored the relationship between the mechanisms of spatial and feature-based attention. We manipulated spatial attention using a precue and feature-based attention through distractor-to-target similarity on a trial-by-trial basis. Reaction times verified that the spatial cue interacted with feature-based attention such that when present, interference from highly target-similar distractors was attenuated. The EEG results showed that the RT data reflected the degree of alpha activity lateralization in posterior channels after target presentation. Poststimulus alpha power was higher contralateral to distractors compared with targets for all cued trials irrespective of distractor similarity, but occurred on uncued trials only with low-similarity distractors. That is, significant poststimulus alpha lateralization occurred when there was a valid spatial cue irrespective of distractor competition, but also when distractor competition was weak and there was no spatial cue. This indicates that alpha lateralization indexes successful target processing (and distractor suppression) similarly when success is due to spatial cueing or low distractor competition. Moreover, the strength of alpha lateralization correlated with individual differences in the degree of distractor interference, suggesting that alpha activity is a general index for the strength of attentional competition between lateralized stimuli.

A lateralization of alpha activity arises because different spatial locations are processed by different hemispheres. In other words, the modulations in alpha activity work as a spatial filter on incoming information. When a target can be detected only based on color during the current experiment, the presence of lateralized alpha activity indicates that the same mechanism used during spatial selection is recruited to enhance target processing after feature-based selection. Finding lateralized alpha during feature-based selection is novel evidence that spatial attention is recruited to protect target processing.

If alpha lateralization reflects attentional processing, one might expect its onset to coincide with the latency of event-related-potential (ERP) components known to reflect attentional selection (Luck and Hillyard, 1994). For example, the N2pc is an ERP component related to lateralized attention that usually occurs 200–300 ms after target presentation. Alpha lateralization in this study was found to begin 350 ms after target presentation for cued trials and 500 ms for uncued trials. This suggests that the alpha modulation in our task occurred after initial attentional selection of the target. The slower onset of lateralized alpha in the uncued compared with cued conditions is also consistent with ERP findings that measurements of spatial attention are observed earlier (70–100 ms after target onset, Gomez Gonzalez et al., 1994) than changes due to feature-based attention (150 ms after target onset, Anllo-Vento et al., 1998). One hypothesis for this difference in latency is that the effect of feature-based attention seen at 150 ms may actually reflect processes that guide spatial attention to the target location after the target had been selected based on its color (Öğmen and Breitmeyer, 2006). The slower onset of alpha lateralization in the uncued condition in our study is consistent with this hypothesis and suggests that targets were selected based on feature-based attentional processes that were followed by a shift in spatial attention.

The fact that poststimulus alpha lateralization started after stimulus offset in our experiment implies that the inhibitory effect of alpha acted on lingering visual representations in working memory and not just on initial stimulus processing, as would be present in the pretarget cue-induced changes in alpha. Our results are consistent with a previous study that related poststimulus alpha lateralization to the efficient suppression of irrelevant information in short-term memory (Sauseng et al., 2009). During that experiment, a spatial cue was used to indicate which hemifield contained to-be-remembered items and which contained distractors. Sauseng et al. found that alpha activity contralateral to the to-be-ignored stimuli correlated with the successful suppression of distractors and better recall of items in the cued visual field. Our findings extend this result in showing that post-target alpha lateralization is related to distractor suppression in response to both spatial cues and feature-based selection. In addition, it is possible that in the uncued and high-similarity condition, subjects were forced to rely more heavily on the shape information to distinguish the target from the distractor and that this may have contributed to the delay in ability to select the correct target. Although we cannot know whether subjects sometimes used both shape information in combination with color when color discrimination was difficult, the current results suggest that irrespective of what featural information was used to select the target, alpha lateralization reflected the continued attentional processing of the target, once it was identified and its location selected. It therefore seems that alpha power modulations reflect the gating of relevant information by inhibition of neuronal spiking (Haegens et al., 2011) in response to task-irrelevant information (Jensen and Mazaheri, 2010).

Interestingly, midfrontal theta showed the opposite pattern to alpha lateralization in the uncued conditions: the uncued high-similarity condition showed no significant alpha lateralization, but did show significant midfrontal theta in a later time period; and the low-similarity condition showed the opposite pattern. Midfrontal theta was present when alpha lateralization was absent and distractor competition was strong, suggesting that midfrontal theta reflected late reactive attentional control mechanisms to reject highly similar distractors that compete for attention when earlier posterior alpha did not successfully suppress the distractor. This suggests that posterior alpha and midfrontal theta reflect mechanisms of earlier and later control of attention, respectively. It should be noted that the time period of increased theta-band activity was late compared to typically found in other tasks (Cavanagh et al., 2012; Nigbur et al., 2012; Cohen and Donner, 2013; Cavanagh and Frank, 2014; Van Driel et al., 2015); however, reaction times in our experiment were also slower.

In sum, our results demonstrate that the modulation of alpha activity is not only a preparatory mechanism for predicted target information and distraction, but also serves to protect processing of relevant information after its selection based on spatial or feature-based information.

## Synthesis

Reviewing Editor: Kathryn Jeffery, University College London<br />Decisions are customarily a result of the Reviewing Editor and the peer reviewers coming together and discussing their recommendations until a consensus is reached. When revisions are invited, a fact-based synthesis statement explaining their decision and outlining what is needed to prepare a revision will be listed below. For some manuscripts transferred from JNeurosci, a decision is made to accept the manuscript without peer review and a brief statement to this effect will follow.

Abstract: This is written for a general audience and should start with a big-picture statement of the hypothesized role of alpha in distractor suppression, and why this is important to understand. The hypothesis and its predictions should also be stated here. Results and conclusion concerning theta band should also be included in the abstract.

l26 - daily-life does not need a hyphen

l31 ipsilateral should be ipsilaterally

l43 examined if should be examined whether

l47 begs the question should be raises the question

ll48-50 This sentence is hard to understand

ll53-55 "If mechanisms of feature-based selection recruit spatial attention once a target is identified, 54 then we might expect the presence of lateralized alpha even when a target can only be selected based 55 on color." The reason needs explaining for the non-specialist

l79 The inclusion of a single left-handed participant needs justifying given that left-handers show different lateralization, and this was a study on lateralized phenomena. The participant arguably should be excluded.

l102 "the cue was uninformative" - can you elaborate here on what this means, as the reader has difficulty visualizing without having encountered teh detailed methodology

Statistics throughout need to be reported properly with all the degrees of freedom etc. t-tests should say whether one-tailed or two.

Figure 2, the y axis should start from zero or indicate the missing part of the axis (preferably the former) - otherwise the scale of the effect is misleading.

Figure 3 Is this for a single subject or combined?

Figure 4 - similarly, is this the population data? There needs to be some statistcal evalutaion of the magnitude and significance of the effec (cumulative prob curve? KS test?)

Figure 5 it is very difficult to see the outlier point

l218 This is the first mention of theta band was done in the analysis section. The role of this band in attentional processes should be stated since the Introduction section. In the discussion section, results of theta band should be discussed in contrast to related literature.

l257 Check: "subsequent analyses analysis"

l266 Parenthesis missing and two many p "(main effect of distractor-type F(3) = 3,249, p = p = 0.030 , p = 0.016."

l268 Parenthesis missing "(no main effect cue-presence F(1) = 1,274, p = 0.277."

l269 Parenthesis missing "type F(3) = 3,175, p = 0.033"

l283 It is not clear what values the t-test is being performed on, is it the peak in the alpha trace?

l329 Legend of Figure 4. "The figures on the left displays the time course of alpha activity for ipsilateral (blue) and contralateral (red) channels." It should be on the right instead of left, shouldn´t it?

Discussion should finish with a big-picture summary of why these results are important in the grand scheme of things.

The reference of Womdeldorf and Everling (2015) is missing in the list of references.

Please check the complete list of references to ensure that all the references of the text are included.
